# Health-related quality of life in hospitalized older patients with versus without prolonged use of opioid analgesics, benzodiazepines, and z-hypnotics: a cross-sectional study

**DOI:** 10.1186/s12877-020-01838-8

**Published:** 2020-10-23

**Authors:** Socheat Cheng, Tahreem Ghazal Siddiqui, Michael Gossop, Knut Stavem, Espen Saxhaug Kristoffersen, Christofer Lundqvist

**Affiliations:** 1 Health Services Research Unit (HØKH), Akershus University Hospital, PO Box 1000, 1478 Lørenskog, Norway; 2 Institute of Clinical Medicine, Campus Ahus, Faculty of Medicine, University of Oslo, Lørenskog, Norway; 3 National Addiction Centre, Institute of Psychiatry, Psychology and Neuroscience, King’s College London, London, UK; 4 Department of Pulmonary Medicine, Akershus University Hospital, Lørenskog, Norway; 5 Department of General Practice, Institute of Health and Society, University of Oslo, Oslo, Norway; 6 Department of Neurology, Akershus University Hospital, Lørenskog, Norway

**Keywords:** Medication safety, Prescription drug abuse, Geriatric patients, Opioids, Z-drugs, Benzodiazepines, Patient-centered care, Old age

## Abstract

**Background:**

Central nervous system depressant medications (CNSDs) such as opioid analgesics and sedative-hypnotics are commonly prescribed to older patients for the treatment of chronic pain, anxiety and insomnia. Yet, while many studies reported potential harms, it remains unknown whether persistent use of these medications is beneficial for older patients’ self-reported health-related quality of life (HRQoL). The present study clarified this knowledge gap through comparing HRQoL of hospitalized older patients with versus without using CNSD drugs for ≥4 weeks. Moreover, we explored the relationship between such use and HRQoL, adjusting for the effects of polypharmacy, comorbidity burden and other clinically relevant covariates.

**Methods:**

The study was cross-sectional and included 246 older patients recruited consecutively from somatic departments of a large regional university hospital in Norway. We defined prolonged CNSD use as using opioids, benzodiazepines and/or z-hypnotics for ≥4 weeks. Patients’ self-reported HRQoL were measured with scales of the EuroQol EQ-5D-3L instrument. Data analyses were mainly descriptive statistics and regression models.

**Results:**

Patients with prolonged use of CNSDs reported lower scores on both EQ-5D index and EQ VAS compared with those without such use (*p* < 0.001). They had higher odds of having more problems performing usual activities (OR = 3.37, 95% CI: 1.40 to 8.13), pain/discomfort (OR = 2.06, 95% CI: 1.05 to 4.04), and anxiety/depression (OR = 3.77, 95% CI: 1.82 to 7.82).

In multivariable regression models, there was no significant association between prolonged CNSD use and HRQoL when including pain as a predictor variable. In models not including pain, CNSD use was strongly associated with HRQoL (adjusted for sociodemographic background, polypharmacy, comorbidity, anxiety and depressive symptoms, regression coefficient − 0.19 (95% CI, − 0.31 to − 0.06).

**Conclusions:**

Older patients with prolonged CNSD use reported poorer HRQoL. They also had more pain and higher depression scores. Prolonged use of CNSDs was not independently associated with higher HRQoL.

## Background

Health-related quality of life (HRQoL) is a subjective and multidimensional construct, which can be conceptualized through reflecting on a patient’s views, experiences or expectation of his or her own physical, mental, social and functional health [1]. Older people may have reduced HRQoL due to age-related progressive decline in physiological reserve and functional capacity [2]. Apart from multi-morbidity, cognitive deficits and psychological distress, inappropriate medication use and poor prescription quality may also undermine HRQoL, through causing drug-induced injuries [3–5].

Chronic pain, anxiety and insomnia are common in old age [6]. Pharmacological therapy of these conditions may involve the use of central nervous system depressant medications (CNSDs) such as opioid analgesics, benzodiazepines and z-hypnotics [7]. Although these medications are frequently prescribed, long-term effectiveness among older people was not shown [8, 9]. Moreover, many studies underline misuse potentials and hazardous consequences of these medications among older patients [10]. Long-term opioid use, for instance, was found to be associated with major adverse effects such as dependence, opioid-induced hyperalgesia, sleep-disordered breathing, hormone dysfunction and immunosuppression [11]. Overuse of benzodiazepines and z-hypnotics likewise was found to be associated with reduced cognitive function, road-traffic accidents, respiratory depression, fractures, falls, and suicide [12, 13].

Research on long-term use of CNSD use in geriatric patients has mostly been restricted to condition-specific outcomes rather than patients’ quality of life as a whole. Also, studies rarely adjust for the effects of multi-morbidity and polypharmacy [14]. Thus, we compared self-reported HRQoL of hospitalized older patients with and without prolonged use of commonly prescribed CNSDs: opioids, benzodiazepines and z-hypnotics.

## Methods

### Study design and setting

This was a cross-sectional, in-hospital study, which recruited patients consecutively between May 2017 and September 2018, from three somatic departments of the Akershus University Hospital: Geriatrics, Internal Medicine and Neurology. The catchment area of the hospital covers approximately 10% of the total population of Norway. Due to an all-covering national health insurance, patients are admitted to the hospital regardless of their socioeconomic background.

### Inclusion and exclusion criteria

Participants were recruited during the first few days of admission based on predefined inclusion and exclusion criteria. The study included hospitalized patients, aged between 65 and 90 years old. The exclusion criteria were Mini-Mental State Examination (MMSE) score ≤ 21 (interpreted as incapacity to give informed consent) [15], pre-existing diagnosis of severe depression, stroke, dementia, psychotic disorders; serious visual or hearing impairment; and insufficient knowledge of Norwegian language, which could bias patients’ responses to self-rated health questions. We precluded participants who were critically ill or in palliative treatment, defined by physicians in the study setting.

### Data collection

After giving informed consent, all eligible participants responded to questionnaires containing questions on sociodemographic background, pain intensity, anxiety, depression and HRQoL. At this stage, the investigators were masked to information on CNSD use as this was registered in the electronic medical record (EMR), which could only be accessed once a written informed consent had been obtained. Having fulfilled this requirement, the EMRs were reviewed to document the use of CNSDs (types, duration, and use patterns) and comorbidity burden.

### Variables and data sources

#### Use of central nervous system depressants (CNSDs)

We defined prolonged use of CNSDs as using opioid analgesics, benzodiazepines and/or z-hypnotics for ≥4 weeks continuously up to the recruitment time point, while non-prolonged use was no use or use of these medications for < 4 weeks. Data on this variable were collected through reviewing EMRs. To ensure data accuracy, we cross-checked for consistency of information in the EMR by checking with the patients and GP referral documents.

#### Assessment of health-related quality of life

The EuroQol Group’s EQ-5D-3L instrument was used to collect data on the HRQoL of study participants [16]. This instrument is a self-reported, standardized questionnaire, which is short, widely used and available in more than 170 languages, including Norwegian. It consists of two parts: the EQ-5D descriptive system and the EQ visual analogue scale (EQ VAS). The descriptive system contains five dimensions: mobility, self-care, usual activities, pain/discomfort and anxiety/depression. Each dimension has three levels of severity. Thus, a total of 243 possible health states can be assigned. Individual patients’ health states are converted into an index value (EQ-5D index) which represents how good or bad a patient’s overall HRQoL is, based on the valuation of health states by the general population. An EQ-5D index of 1 is anchored as full health, while 0 represents death. The calculation of EQ-5D index score was performed using the value set for England because values for Norway were not available. The EQ VAS is a 20 cm vertical visual analogue scale (VAS), on which participants record self-perceived health on a 0 to 100 scale, where 0 is “the worst health possible” and 100 “the best imaginable health”. When filling in the EQ-5D questionnaire, participants were asked to refer to their current health status and situation [16].

#### Sociodemographic and clinical variables

Sociodemographic variables included sex, age (years), education (basic, secondary and higher education) and living alone (yes, no). Clinical variables entailed current pain intensity (measured on a 10 cm horizontal VAS) [17], anxiety and depressive symptoms (assessed with the Hospital Anxiety and Depression Scale – HADS) [18], comorbidity burden (defined as the total score of the Cumulative Illness Rating Scale for Geriatrics – CIRS-G) [19], polypharmacy (using ≥5 medications/day) [20]. The HADS is a 14-item questionnaire, containing two subscales: anxiety (7 questions) and depression (7 questions). Optimal cut-off values for diagnosing anxiety and depression in hospitalized older patients using HADS remain to be established [21, 22]. Therefore, we used anxiety and depression scores as continuous variables on 0 to 21 scales, with higher scores indicating more symptoms. The CIRS-G is a validated scale for assessing comorbidity and the burden of chronic illnesses in geriatric patients. It has 14 organ-system categories, scored from 0 to 4, with higher scores indicating increased severity of medical conditions [23]. Data on sociodemographic status, pain intensity, anxiety and depression scores were all collected through a self-completed questionnaire, while data for comorbidity burden were collected through reviewing EMRs.

### Statistical analysis

We compared participants’ characteristics and HRQoL (EQ-5D dimension scores, EQ VAS and EQ-5D index score) using the chi-square test, student’s t-test, or Mann-Whitney U test, as appropriate. We also performed a post-hoc analysis to assess differences in EQ VAS and EQ-5D index scores between patients who used only 1 type versus ≥2 different types of CNSDs as well as among the users of the three listed medication groups.

Initially, we attempted to use ordered logistic regression analyses to assess the association between prolonged use of CNSDs and the five dimensions of the EQ-5D-3L, as they have three levels of severity. The proportional odds assumption was checked using the Brant test and was met for the models with mobility, self-care, pain/discomfort and anxiety/depression as the dependent variables. The model with usual activities as the dependent variable did not satisfy the assumption (Brant tests: *P* < 0.05) [24] and was therefore analyzed with binary logistic regression analysis. For this model, we dichotomized responses into absence (level 1) or presence (level 2 and 3) of problems regarding usual activities. In the assessment of the relationship between prolonged CNSD use and problems regarding mobility, self-care and usual activities, we included the following covariates: sociodemographic background, polypharmacy and comorbidity burden, pain intensity (VAS) and anxiety and depression scores (HADS). To avoid over-adjustment, we dropped the variable pain intensity (VAS) from the model where pain/discomfort (EQ-5D) was the dependent variable as they both measure the same construct. For the same reason, we dropped anxiety and depression scores (HADS) as independent variables when anxiety/depression (EQ-5D) was the dependent variable. The results of ordered logistic regression analysis is interpreted as the odds of being in groups greater than *j* versus those who are in groups less than or equal to *j*, where *j* is the level of the dependent variable. This is constant across all levels of the dependent variable [24]. For example, the proportional odds ratio for the mobility dimension of the EQ-5D-3L represents the odds of being confined to bed (level 3) versus no/some problems in walking about (level 1 and 2), and also the odds of having some problems in walking about/being confined to bed (level 2 and 3) versus no problems in walking about (level 1).

We explored the association between prolonged use of CNSDs and overall HRQoL as assessed by the EQ-5D index in three different models, using hierarchical multiple linear regression analysis. The purpose was to gain a better understanding of the relationship. For Model 1, we adjusted for the effects of covariates: age, sex, education, living alone, polypharmacy and comorbidity burden. In Model 2, we added two extra independent variables to the model: anxiety and depression (HADS) scores, known to affect HRQoL [25]. Further, we created Model 3 by adding the variable pain intensity (VAS) to the Model 2, as we considered pain to be a possible confounder. As the percentage of missing values for EQ-5D-3L was minimal (*n* = 7), complete case analysis was used. Multicollinearity was checked based on the variance inflation factor values and was not detected. Residual diagnostics was performed, and bootstrap confidence intervals were also calculated to confirm the main results. The results are presented with regression coefficient (unstandardized beta coefficients) with 95% confidence interval (CI). Stata SE software, version 15 (StataCorp, College Station, TX, USA) was used for all statistical analyses. We chose a 5% significance level, using two-tailed tests.

### Patient and public involvement

The present study engaged patient representatives, health professionals, and data protection officers in the design, performance, and dissemination of research results. We had regular meetings at the Akershus University Hospital to discuss, update, and provide inputs on practical issues pertaining to the study.

## Results

**Fig. 1:**
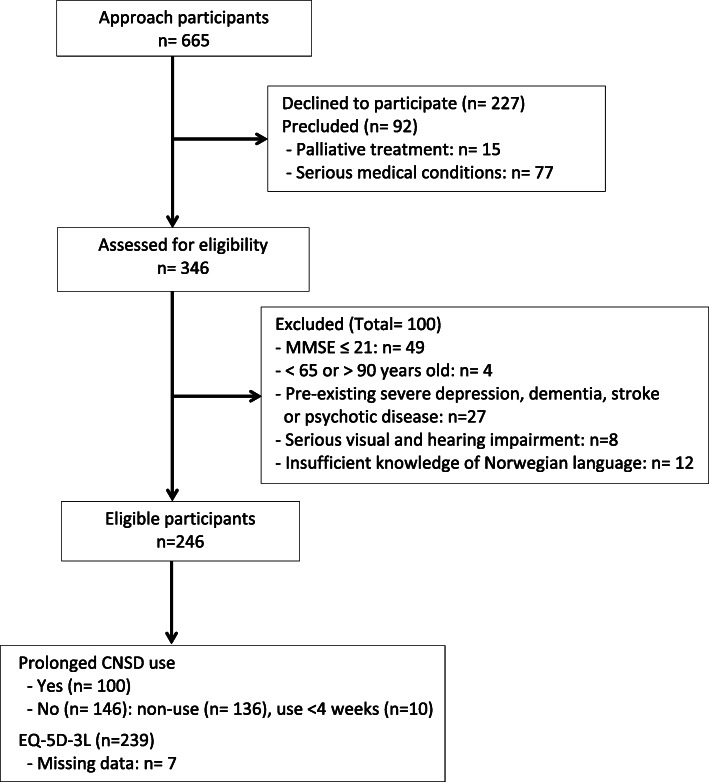
Flow of participants through the study

A total of 665 hospitalized patients aged 65–90 were consecutively approached during the study period. Of these, 227 patients declined to participate, 92 were precluded because of being in a critical medical condition or palliative treatment, and 100 were excluded based on predefined exclusion criteria. Hence, 246 older patients were eligible for the study. A total of 100 participants were prolonged users of CNSDs (Fig. 1); 70% used only one type of CNSDs: z-hypnotics 42%, opioid analgesics 21%, and benzodiazepines 7%. The remaining 30% concurrently used ≥2 different types of CNSDs. Participants with prolonged use of CNSDs comprised a higher proportion of females, were older, had less education, more polypharmacy, more comorbidity and higher pain intensity and depression scores than those without prolonged use (Table 1).

**Table 1:** Characteristics of participants, number (%) unless stated otherwise

	Prolonged CNSD use		
	No (***n*** = 146)	Yes (***n*** = 100)	***p***-value
**Sex**, female	71 (49)	66 (66)	0.009
**Age** in years, mean (SD)	75 (6.4)	78 (6.5)	< 0.001
**Education**			
Secondary education	64 (46)	31 (33)	0.001
Higher education	58 (42)	33 (35)	
**Living alone**	59 (40)	55 (55)	0.03
**Polypharmacy**, ≥5 drugs/day	91 (62)	92 (92)	< 0.001
**Comorbidity burden** (CIRS-G scores), mean (SD)	4.6 (2.2)	7.7 (2.7)	< 0.001
**Hospital anxiety and depression scale**			
Anxiety score (HADS-A), median (IQR)	4 (1 to 6)	4 (2 to 8)	0.17
Depression score (HADS-D), median (IQR)	3 (1 to 6)	4 (2 to 7)	< 0.001
**Pain intensity** (VAS in centimeters), median (IQR)	0.7 (0.03 to 2.7)	2.9 (0.5 to 6.1)	< 0.001

*Abbreviations:CNSD* Central nervous system depressant drugs, *CIRS-G* Cumulative Illness Rating Scale-Geriatrics, *VAS* Visual analogue scale, *SD* Standard deviation, *IQR* Interquartile range

### Comparison of HRQoL between patients with and without prolonged CNSD use

Patients with prolonged use of CNSDs reported greater problems concerning mobility, self-care, usual activities, pain/discomfort and anxiety/depression (dimensions of the EQ-5D) than those without prolonged use. They also had lower EQ VAS scores, mean 50 (SD 17) than those without such use, mean 62 (SD 23), *p* < 0.001 (Table 2). Patients with prolonged CNSD use reported lower EQ-5D index scores (median 0.19, IQR: 0.002 to 0.69) than those without (median 0.73, IQR: 0.30 to 0.81), *p* < 0.001. In our post-hoc analyses, we did not find differences in EQ VAS and EQ-5D index scores between patients who used just 1 type and those concurrently used ≥2 types of CNSDs. Also, EQ VAS and EQ-5D index scores of opioid, benzodiazepine and z-hypnotic users did not differ (data not shown).

**Table 2:** Health-related quality of life of patients with versus without prolonged CNSD use

	Prolonged CNSD use		
	No (***n*** = 146)	Yes (***n*** = 100)	***p***-value
**Dimensions of HRQoL**, n (%)			
**Mobility**			
No problems in walking about	60 (42)	16 (17)	< 0.001
Some problems in walking about	59 (41)	52 (54)	
Being confined to bed	24 (17)	28 (29)	
**Self-care**			
No problems with self-care	109 (76)	50 (52)	< 0.001
Some problems with self-care	28 (20)	40 (42)	
Being unable to wash or dress myself	6 (4)	6 (6)	
**Usual activities**			
No problems with performing my usual activities	77 (54)	16 (17)	< 0.001
Some problems with performing my usual activities	45 (31)	61 (63)	
Being unable to perform my usual activities	21 (15)	19 (20)	
**Pain/discomfort**			
No pain or discomfort	48 (34)	19 (20)	0.02
Moderate pain or discomfort	76 (53)	53 (55)	
Extreme pain or discomfort	19 (13)	24 (25)	
**Anxiety/depression**			
Not being anxious or depressed	96 (67)	40 (42)	< 0.001
Being moderately anxious or depressed	44 (31)	47 (49)	
Being extremely anxious or depressed	3 (2)	9 (9)	
**EQ VAS**, mean (SD)	62 (23)	50 (17)	< 0.001
**Global score of HRQoL (EQ-5D index)**, median (IQR)	0.73 (0.30 to 0.81)	0.19 (0.002 to 0.69)	< 0.001

*Abbreviations*: *CNSDs* Central nervous system depressant drugs; *VAS* Visual analogue scale, *SD* Standard deviation, *IQR* Interquartile range

### Association between prolonged use of CNSDs and HRQoL in multivariable analyses

#### Dimensions of HRQoL

In multiple logistic regression analyses (Table 3), we found that the odds of having extreme versus moderate/no pain or discomfort, and also the odds of having moderate/extreme versus no pain or discomfort for patients with prolonged CNSD use were about two times higher than that of those without such use (OR = 2.06, 95% CI 1.05 to 4.04). Patients with prolonged CNSD use were four times more likely than those without prolonged use to be extremely versus moderately/not anxious or depressed, or moderately/extremely versus not anxious or depressed (OR = 3.77, 95% CI: 1.82 to 7.82). Also, using CNSDs for ≥4 weeks was associated with increased odds of having problems performing usual activities (OR = 3.37, 95% CI: 1.40 to 8.13). In addition, we found that polypharmacy was associated with problems performing usual activities and mobility. The odds of being confined to bed versus no/some problems in walking about, and also the odds of being confined to bed/some problems in walking about versus no problems in walking about for patients with polypharmacy were 3.58 times higher than that of those without polypharmacy (OR = 3.58, 95% CI: 1.77 to 7.22). The odds of being unable to perform usual activities versus some/no problems with performing usual activities, and also the odds of being unable/some problems versus no problems with performing usual activities for patients with polypharmacy were 2.73 times higher compared to those without polypharmacy (OR = 2.73, 95% CI: 1.28 to 5.84).

**Table 3:** Multiple logistic regression analyses of the association between prolonged use of CNSDs and the five dimensions of HRQoL

Independent variable	EQ-5D dimensions				
	Mobility^a^ OR (95%CI)	Self-care^a^ OR (95%CI)	Usual activities^b^ OR (95%CI)	Pain/discomfort^a^ OR (95%CI)	Anxiety/depression^a^ OR (95%CI)
Prolonged CNSD use, yes versus no	1.22 (0.61 to 2.44)	1.49 (0.68 to 3.25)	3.37 (1.40 to 8.13)	2.06 (1.05 to 4.04)	3.77 (1.82 to 7.82)
Polypharmacy, ≥5 versus < 5 drugs/day	3.58 (1.77 to 7.22)	1.67 (0.71 to 3.95)	2.73 (1.28 to 5.84)	1.65 (0.86 to 3.17)	0.55 (0.27 to 1.10)
Comorbidity burden (CIRS-G total score)	1.08 (0.97 to 1.20)	1.05 (0.93 to 1.19)	1.02 (0.87 to 1.19)	1.08 (0.96 to 1.21)	0.96 (0.85 to 1.07)
Anxiety score (HADS-A)	0.96 (0.87 to 1.06)	0.94 (0.84 to 1.05)	1.01 (0.90 to 1.15)	1.11 (1.01 to 1.22)	N/A
Depression score (HADS-D)	1.15 (1.04 to 1.28)	1.18 (1.05 to 1.33)	1.16 (1.01 to 1.33)	1.00 (0.90 to 1.11)	N/A
Pain intensity (VAS)	1.17 (1.06 to 1.29)	1.19 (1.06 to 1.33)	1.20 (1.04 to 1.37)	N/A	1.05 (0.95 to 1.17)

Note: all models are adjusted for age, sex, education and living alone. ^a^: Ordered logistic regression, ^b^: Binary logistic regression

*Abbreviations*: *OR* Odds ratio, *CNSDs* Central nervous system depressant drugs, *HADS–D/A* Hospital anxiety and depression sub-scales, *VAS* Visual analogue scale, *N/A* Not applicable, *CI* Confidence interval, *CIRS-G* Cumulative illness rating scale for Geriatrics

#### Global score of HRQoL (EQ-5D index)

In hierarchical multiple linear regression analyses, we found that older patients who had used CNSDs for ≥4 weeks had lower EQ-5D index scores, regression coefficient − 0.20 (95% CI: − 0.32 to − 0.08, *p* = 0.002) than that of those without such use, adjusted for age, sex, education, living alone, polypharmacy and comorbidity burden (Table 4, Model 1). In Model 2, when adding adjustment for anxiety scores (HADS-A) and depression scores (HADS-D) to the Model 1, the regression coefficient barely changed, − 0.19 (95% CI: − 0.31 to − 0.06, *p* = 0.004). However, in Model 3 (adding the variable pain intensity to the Model 2), the coefficient for prolonged CNSD use was − 0.08 (95% CI: − 0.21 to 0.05, *p* = 0.24), while pain intensity − 0.06 (95% CI: − 0.08 to − 0.04, *p* < 0.001), and depression score − 0.02 (95% CI: − 0.04 to − 0.001, *p* = 0.04) were statistically significant. Polypharmacy was associated with reduced EQ-5D index in all models, with the regression coefficient ranging from − 0.12 to − 0.15. Residual diagnostics was performed, and even though some minor deviation from model assumptions were observed, the bootstrap-based inference did not change the results.

**Table 4:** Multiple linear regression analyses of the association between prolonged use of CNSDs and the global score of HRQoL (EQ-5D index)

Independent variable	Model 1		Model 2		Model 3	
	Regression coefficient (95% CI)	***p***-value	Regression coefficient (95% CI)	***p***-value	Regression coefficient (95% CI)	***p***-value
Prolonged CNSD use	−0.20 (−0.32 to −0.08)	0.002	−0.19 (− 0.31 to − 0.06)	0.004	− 0.08 (− 0.21 to 0.05)	0.24
Polypharmacy, ≥5 versus < 5 drugs/day	−0.15 (− 0.27 to − 0.04)	0.01	−0.15 (− 0.26 to − 0.03)	0.01	−0.12 (− 0.23 to − 0.01)	0.04
Comorbidity burden (CIRS-G total score)	−0.01 (− 0.04 to 0.01)	0.21	−0.01 (− 0.03 to 0.01)	0.27	−0.01(− 0.03 to 0.01)	0.22
Anxiety score (HADS-A)			−0.02 (− 0.03 to 0.003)	0.10	− 0.01 (− 0.03 to 0.01)	0.20
Depression score (HADS-D)			−0.02 (− 0.04 to 0.002)	0.08	− 0.02 (− 0.04 to − 0.001)	0.04
Pain intensity (VAS), per cm					−0.06 (− 0.08 to − 0.04)	< 0.001
Adjusted R-squared	0.13		0.19		0.34	

Note: all models are adjusted for age, sex, education and living alone

*Abbreviations*: *CNSD* Central nervous system depressant drugs, *HADS–D/A* Hospital anxiety and depression sub-scales, *VAS* Visual analogue scale, *CI* Confidence interval, *CIRS-G* Cumulative illness rating scale for Geriatric

## Discussion

This study has shown that older patients who had used CNSDs ≥4 weeks had poorer HRQoL than those with non-use or use < 4 weeks, but also that the presence of pain as a confounder modified this finding. Patients using CNSDs reported lower EQ VAS and EQ-5D index scores and had higher odds of having impairment in several dimensions of the EQ-5D (usual activities, pain/discomfort and anxiety/depression). There was no association between CNSD use and higher HRQoL. We found an inverse relationship between prolonged CNSD use and older patients’ overall HRQoL as assessed with EQ-5D index, in a model adjusted for sociodemographic variables, polypharmacy, comorbidity burden, anxiety and depressive symptoms. In an additional regression model which also included pain, this relationship was no longer significant. When pain was also included in the model, only pain, depression, and presence of polypharmacy were significantly associated with lower HRQoL.

A strength of this study is that it focused specifically on HRQoL of older patients with versus without prolonged use of CNSDs. Moreover, we had the opportunity to adjust for clinically important and often-neglected variables such as comorbidity burden and polypharmacy. Our study has some limitations. It was not designed for and did not have sufficient power to study associations with subgroups of CNSD separately. However, our focus has been the combined effect of the whole CNSD medication group as these drugs are commonly prescribed together for older patients. Thus, we suggest that our findings reflect a pragmatic real life situation, which may, in fact, be missed in studies focusing on separate medications. The use of a cross-sectional design precludes us from inferring direction of causation. Possibly, prolonged use of CNSDs leads to health problems such as difficulty performing usual activities, pain/discomfort, and anxiety/depression. However, the reverse is also possible as rebound anxiety and opioid-induce hyperalgesia may occur [26–28]. Future longitudinal or intervention studies should clarify this. The study may be still subject to confounding by indication as we have not assayed any parameters related to sleep and have no possibility to reconstruct the design. However, the observed associations were examined with multivariable regression models, adjusting for variables which are clinical indications for CNSD therapy such as pain, anxiety and depression. The effect of confounding by indication was therefore partly addressed. We calculated EQ-5D index scores based on the value set for England, as a tariff was not available for Norway. Using a consecutive hospital-based sample limits the generalizability of the study findings. However, our sample constitutes patients admitted to clinical wards regardless of their socioeconomic background and for a variety of health problems, and as such should be representative for hospital populations of older patients, admitted to geriatric, general internal medicine and neurology departments.

Our study showed that patients with prolonged CNSD use reported poorer HRQoL than those without prolonged use (Table 2). As the most severely afflicted patients were excluded, mainly for ethical reasons, our results may be somewhat biased towards milder cases. This may suggest that, could the most severe cases have been included, the effect size may have been even larger. A systematic review, though not focused on older patients, pointed out that there is only weak evidence that long-term opioid therapy may improve HRQoL [29].

The finding that depression scores were higher among prolonged CNSD users than those without such use ties in well with previous studies. Both randomized and non-randomized studies have shown that the use of benzodiazepines and z-hypnotics may increase the risk of subsequent depression [30, 31]. Depression may worsen pain intensity and erodes opioid analgesic potency, challenging the treatment of chronic pain. Depressed patients are also more likely to initiate and continue using opioids than those without depression [32, 33]. In the present study, depression was negatively associated with patients’ overall HRQoL, as assessed with the EQ-5D index, in line with other studies [34, 35].

Our study showed that polypharmacy was associated with impairment in two dimensions of HRQoL. Also, polypharmacy was inversely associated with patients’ overall HRQoL, as assessed with the EQ-5D index score in all the three multiple linear regression models. These findings are consistent with some previous studies [36, 37].

This study also indicated that patients with prolonged CNSD use had more intense pain then those without such use, and that pain intensity was associated with lower HRQoL. These results support previous studies [38, 39]. In two of the hierarchical multiple linear regression models, prolonged CNSD use was associated with the EQ-5D index. However, when adding the variable pain intensity (VAS) to the model, the observed association between prolonged CNSD and the EQ-5D index was no longer statistically significant, accompanied by a 15% increase in the adjusted R-squared value. Hence, pain intensity may better explain the variance in EQ-5D index, beyond those variables in the Model 2. It is possible that the observed association between prolonged CNSD use and EQ-5D index (Model 2) was confounded by pain intensity, as patients with prolonged CNSD use had more pain than the reference group, and that pain intensity had a negative relationship with the EQ-5D index. Alternatively, pain intensity may operate as an intermediate step between prolonged CNSD use and HRQoL.

### Clinical implication

It was unknown whether persistent use of CNSD medications was beneficial for older patients’ HRQoL. This study indicates that older patients with prolonged CNSD use have poorer HRQoL and higher odds of having impairment in multiple dimensions of HRQoL than those without such use. After adjusting for current pain in multivariable analyses, an approach which has been discussed due to the complex nature of pain as a symptom [40], prolonged use of CNSDs is not independently associated with higher HRQoL.

## Conclusions

Older patients with prolonged use of CNSDs do not have better HRQoL than those without such use. The presence of pain as well as anxiety and depression seem to be important predictors and our study emphasizes the importance of considering these factors carefully in assessing the indications for long-term CNSD treatment among older patients. Further prospective longitudinal studies are necessary for assessing direction of causality.

## Data Availability

The datasets generated and/or analyzed during the current study are available from the corresponding author on reasonable request.

## Abbreviations

CNSD: Central nervous system depressant drugs
HRQoL: Health-related quality of life
HADS–D/A: Hospital anxiety and depression sub-scales
VAS: Visual analogue scale
CIRS-G: Cumulative illness rating scale for Geriatrics
OR: Odd ratio
CI: Confidence interval
SD: Standard deviation
IQR: Interquartile range
